# Host-Associated Bacterial Communities Vary Between *Daphnia galeata* Genotypes but Not by Host Genetic Distance

**DOI:** 10.1007/s00248-022-02011-x

**Published:** 2022-04-29

**Authors:** Amruta Rajarajan, Justyna Wolinska, Jean-Claude Walser, Stuart R. Dennis, Piet Spaak

**Affiliations:** 1 Department of Aquatic Ecology, Swiss Federal Institute of Aquatic Science and Technology (Eawag), Dübendorf, Switzerland; 2 Department of Evolutionary and Integrative Ecology, Leibniz Institute of Freshwater Ecology and Inland Fisheries (IGB), Berlin, Germany; 3 Department of Biology, Chemistry, Pharmacy, Institut Für Biologie, Freie Universität Berlin (FU), Berlin, Germany; 4 Genetic Diversity Centre, ETH Zürich, Zürich, Switzerland

**Keywords:** 16S rDNA, Bacteria, Cladocera, Microbiome, Zooplankton

## Abstract

Host genotype may shape host-associated bacterial communities (commonly referred to as microbiomes). We sought to determine (a) whether bacterial communities vary among host genotypes in the water flea *Daphnia galeata* and (b) if this difference is driven by the genetic distance between host genotypes, by using *D. galeata* genotypes hatched from sediments of different time periods. We used 16S amplicon sequencing to profile the gut and body bacterial communities of eight *D. galeata* genotypes hatched from resting eggs; these were isolated from two distinct sediment layers (dating to 1989 and 2009) of a single sediment core of the lake Greifensee, and maintained in a common garden in laboratory cultures for 5 years. In general, bacterial community composition varied in both the *Daphnia* guts and bodies; but not between genotypes from different sediment layers. Specifically, genetic distances between host genotypes did not correlate with beta diversity of bacterial communities in *Daphnia* guts and bodies. Our results indicate that *Daphnia* bacterial community structure is to some extent determined by a host genetic component, but that genetic distances between hosts do not correlate with diverging bacterial communities.

**Supplementary Information:**

The online version contains supplementary material available at 10.1007/s00248-022-02011-x.

## Introduction

Bacterial communities that reside within animal hosts are a powerful force influencing the biology of their hosts. The extent of bacterial communities’ influence on host life history, physiology, and behavior has long been a subject of investigation. Across the animal kingdom, host-associated bacteria may play a nutritional role [1], promote developmental processes in the host [2], and aid hosts in acclimating to ecological stressors such as low temperatures [3] and colonization resistance against invading natural pathogens [4]. Associated bacterial communities are also implicated in mediating their hosts’ adaptation to selection pressures through their variation across host genotypes [5, 6]. Host-associated microbes may be vertical transmitted directly through a variety of specialized mechanisms involving depositing microbes in or on the egg [7] or indirectly by parents altering the environment offspring are exposed to [8].

Despite the documented benefits of host-associated microbiota in a wide range of animal hosts, signatures of phylosymbiosis between host species and host-associated microbial community structure vary between different groups of animals. For instance, there is almost no association between microbial communities and avian host species or genotype structure [9] while marine invertebrate species show varying degrees of host genetic versus environmental forces influencing the structure of their associated microbial communities [10]. Genome-wide association studies in humans and mice have reported various genomic loci playing significant roles in host-associated microbial community composition [11, 12], which are generally involved in either host immunity or various lipid or carbohydrate metabolism pathways. In the aquatic snail *Biomphalaria glabrata*, genotypic variation at a single locus involved in pathogen recognition is responsible for an altered host-associated bacterial community [13]. However, these studies generally report varying degrees of host genetic versus environmental (e.g., diet) influence on host-associated microbial community structures. Hence, more studies in more diverse animal models are required to elucidate the role of deterministic forces such as host genetics in host-associated microbial community structure to better understand its role in host ecology and evolution [14].

The freshwater crustacean *Daphnia* is a well-established model system in ecology and evolutionary biology and is a particularly compelling system to investigate host bacterial community dynamics, both within an individual host and on host populations [15]. Studies in *Daphnia magna* indicate that host-associated bacterial communities are generally required for survival, reproduction, and nutrition [16]. *Daphnia-*associated bacteria also influence ecologically relevant traits such as tolerance to cyanobacteria [17, 18], host embryonic development [19], and overall ecological success [20]. The community composition of gut bacteria in *D. magna* is influenced by both the host genotype and by a variation in environmental bacteria [21, 22]. However, the extent of host genotype influence on *Daphnia* bacterial communities is debated, with a laboratory study revealing no variation in whole *D. magna* bacterial communities by host genotype [23] and a recent mesocosm study showing no variation by host genotype in *D. magna* gut bacterial communities raised in natural lake water [24].

Paleo-genetic reconstruction studies suggest that the genetic architecture of *Daphnia* populations varies by sediment depth and reflects responses to environmental stressors over time [27]. The bacterial communities associated with *Daphnia*-resting eggs deposited in the sediment have a beneficial effect on the survival of *Daphnia* hatched from those eggs, and may be required for the establishment of clonal lines [19]. However, no studies so far have utilized naturally occurring genetic variation among *Daphnia* across sediment depth to address host genotypic variation as a determinant of composition of bacterial communities in *Daphnia*. Also, studies investigating variation of the bacterial community associated specifically with the *Daphnia* tissue excluding the gut (e.g., filtering apparatus) are few, though these other associated bacteria may play a role in ecosystem level processes such as transfer of dissolved organic matter across the food web [28].

Here we investigate whether *Daphnia galeata* clonal lineages harbor genotype-specific bacteria after being reared in common garden in a laboratory setting for 5 years. We sequenced the bacterial communities associated with eight *D. galeata* genotypes hatched from two distinct sediment layers (1989 or 2009) of lake Greifensee, corresponding to different stages of reoligotrophication in that lake. *Daphnia* population structure changed considerably with eutrophication and reoligotrophication in Greifensee [27]. Genotypes isolated from the same sediment layer are genetically more similar than those from another sediment layer based on whole-genome sequencing data [29]. We determined whether bacterial communities of the (a) gut and (b) remaining body tissue of *D. galeata* differed between genotypes and sediment layers from which they were hatched (2 sediment layers × 4 genotypes) and further tested for a correlation between genetic distance among *Daphnia* genotypes and dissimilarity in their bacterial community composition. We also compared host-associated bacterial communities to those in the medium in which the *Daphnia* were reared to empirically validate the common garden used in this study. We hypothesized that *Daphnia*-associated bacterial communities would vary significantly by (a) the host genotype and (b) the sediment layer of origin, since sediment layers in our study represent distinct genetic clusters of *Daphnia*. Furthermore, we did not expect bacterial communities of water medium the *Daphnia* were kept in to differentiate between *Daphnia* genotypes or between sediment layers of origin.

## Material and Methods

### *Daphnia galeata* Culturing Conditions (for Bacterial Community Sequencing Experiment).

The eight *D. galeata* genotypes in this study each originated from resting eggs taken from a single sediment core collected from Greifensee (N 47° 20′ 41″, E 8° 40′ 21″) on 16 December 2014 (see also [29]). In the same week, intact resting eggs were collected from two separate sediment layers (corresponding to years 1989 and 2009; the sediment core dating method is described in [30]) and hatched simultaneously in 6-well plates containing Greifensee lake water filtered through a 0.45-µm mesh. Resting eggs were placed in separate wells (but not in a sterile environment). After hatching, the genotypes were maintained as clonal lineages in identical laboratory conditions for 5 years. Standard conditions were: animals were maintained at 12 °C in 100-mL filtered Greifensee lake water, routinely fed with the green algae *Acutodesmus obliquus* (formerly *Scenedesmus obliquus*) grown in a chemostat in WC medium [31] and diluted in filtered lake water before feeding. The amount of added food (three times a week) corresponded to 0.9 mg C/L per 10 animals. Medium was refreshed every 5–6 weeks. In preparation for an experiment, four genotypes (GR_020, GR_023, GR_024, GR_025) from the sediment layer dating to 1989 and four (GR_052, GR_053, GR_054, GR_055) from the layer 2009 were moved to 20 °C in October 2019, split into three replicate lines and maintained simultaneously for the experiment (see below).

### Experimental Setup

Seven to eight females with eggs (8 genotypes × 3 replicates = 24 populations) were maintained together in 200-mL medium and fed 1.5 mg C/L daily, with medium change every alternate day. Twenty to 27 juveniles (experimental animals) produced by the females were moved to fresh 200-mL medium and fed 3 mg C/L with a medium change on alternate days. Jars with experimental animals were assayed twice a day for free-swimming juveniles of the next generation. Adult *Daphnia* were moved to fresh medium (~ 24 h after the first appearance of two to three free-swimming juveniles) to allow loosely associated-bacteria and food particles to diffuse away from the *Daphnia* before dissection. Twenty adult *Daphnia* per replicate were then dissected 52 ± 3 h after the first appearance of juveniles (or 26 ± 2 h after transfer to fresh medium). Two different batches of filtered lake water were used as *Daphnia* medium during the experiment.

### Preparation of DNA Material

For gut samples, 20 *Daphnia* were dissected under a stereo microscope, each in individual droplets of nuclease-free water using sterilized forceps, and extracted guts were immediately moved to a 20-µL droplet of nuclease-free water. This pool of 20 guts was then transferred to a 1.5-mL microcentrifuge tube. For body samples, the remaining *Daphnia* tissue after the extraction of guts was pooled into a separate 1.5-mL microcentrifuge tube. Forceps were flamed between individual dissections to minimize cross-contamination between gut and body samples. For medium samples, 200 mL *Daphnia* medium from which the experimental animals were collected prior to dissection was filtered through a 0.22-µm filter using a sterile syringe. The filter was transferred to a 2-mL microcentrifuge tube. All samples were immediately stored at − 20 °C until further processing. Preparation of DNA material was done on nine non-consecutive days, and the order of processing was randomized across genotypes and replicates.

### Bacterial Community Profiling

DNA was extracted using the Qiagen Blood & Tissue kit (Cat #69,506). Briefly, all samples were lysed at 56 °C for 4 h after which the recommended protocol for DNA extraction provided by the manufacturer of the Qiagen Blood & Tissue kit was followed for *Daphnia* gut and body samples. Modifications to the protocol were made in extraction reagent volumes for the medium samples according to [32] in order to maximize DNA recovery. All samples were eluted in 40 µL kit elution buffer for 20 min.

A nested PCR approach was done due to samples being of low biomass. Universal 16S primers 27F (5′-AGAGTTTGATCMTGGCTCAG-3′) and 1492R (5′-GGTTACCTTGTTACGACTT-3′) were used to amplify the full-length 16S gene with the following cycling conditions 94 °C—30 s; 50 °C—45 s; 68 °C—90 s; 30 cycles [33, 34]. Amplified products were purified using the QiaQuick PCR purification kit (Cat# 28,106) before standard library preparation and amplicon metagenomics of the V3-V4 region using primers 515F (5′-GTGCCAGCMGCCGCGGTAA-3′) and 806R (5′-GGACTACHVGGGTWTCTAAT-3′). Library preparation and sequencing was done by Novogene UK (www.novogene.com Cambridge, UK).

### Pre-Processing of 16S Sequencing Reads

Sequencing resulted in ~ 8.3 M reads (minimum = 55,804, maximum = 137,685 per sample). Raw reads were trimmed, quality filtered, and chimeras were removed. Amplicon sequence variants were clustered using UPARSE [35], denoised into Zero-radius OTUs (ZOTUs) based on 97% sequence similarity using UNOISE3 [36] and annotated using the non-Bayesian SINTAX classifier [37] and the Silva database [38]. Eight ZOTUs (4 of unidentified phylum, 4 chloroplasts) were filtered from the dataset. Samples were rarefied to an even depth of 55,000 reads. The rarefaction step resulted in the removal of one ZOTU, resulting in a total of 432 ZOTUs in the dataset. Whole-genome sequencing data processing of the eight genotypes in this study was performed separately [29].

### Biodiversity Measures and Statistical Testing

All analyses were carried out in R v4.0.2 using the phyloseq package [39]. First, we investigated variation in bacterial community beta diversity between sediment layers of origin and among *Daphnia* genotypes. A two-way PERMANOVA of the β-diversity metric Weighted Unifrac distance was performed with genotype nested within sediment layer using the *adonis* function in the vegan package [40]. The Weighted Unifrac distance captures differential relative abundance as well as phylogenetic relatedness of ZOTUs. This was done separately for each sample type (i.e., *Daphnia* gut, body, and medium), as we were primarily interested in differences between genotypes and sediment layers. Finally, the *capscale* function was used to estimate the percentage of variation explained by PERMANOVA models.

Second, we tested for a correlation between Weighted Unifrac distances between bacterial communities (using the above 16S data) and genetic distances between host genotypes using distances based on 41,771 SNPs from *Daphnia* whole-genome sequencing data [29]. A Mantel test was performed between the dissimilarity matrices, separately for *Daphnia* gut and body bacterial communities using the *mantel* function of the vegan package (Spearman’s rho, 9999 permutations). Hierarchical clustering was performed on Weighted Unifrac distance of *Daphnia* gut and body-associated bacteria as well as genetic distances between host genotypes using the *hclust* function (method = Ward.D2) and customized using the dendextend package [41]. We also identified ZOTUs which could be indicative of specific *Daphnia* genotypes based on their abundance distributions using the Indicspecies package in R, separately for each tissue type. For this, we used the *signassoc* function (two-tailed test, 9999 permutations, corrected for multiple comparisons using the Sidak method) [42].

Third, we investigated the differential abundance of the dominant classes of bacteria in *Daphnia* tissues and medium between sediment layers. ZOTUs were aggregated at the class level using the *tax_glom* function. The bacterial classes that constituted < 1% of counts in the dataset (unless they were present in all samples) were classified as “Other” for visual representation and identification of differential abundance among dominant classes using the *aggregate_rare* function of the microbiome package (an extension of phyloseq). The DESeq2 package (Wald test) was used on agglomerated classes to identify differentially abundant classes [43]. Relative abundances were compared between sediment layers separately for each sample type (4 genotypes × 3 replicates = 12 samples per sediment layer). Then, relative abundances of major bacterial classes were also compared between sample types; i.e., across *Daphnia* gut, body, and medium (8 genotypes × 3 replicates = 24 samples per sample type). We then used the indicator species analysis described above to identify taxa that associate specifically with sample type.

Finally, alpha diversity metrics ZOTU richness and the Shannon Index were calculated for all samples using the *estimate_richness* function. ANOVAs were carried out for both alpha diversity measures, for all sample types together (three-way ANOVA with sediment layer, sample type, and genotype nested within sediment layer). This was followed by posthoc TukeyHSD tests to identify pairwise differences among (a) genotypes and (b) sample types (“sediment layer” was excluded from the posthoc test since it was not significant in the main test, see Results).

## Results

### Beta Diversity of Bacterial Communities Between *Daphnia* Genotypes

**Fig. 1:**
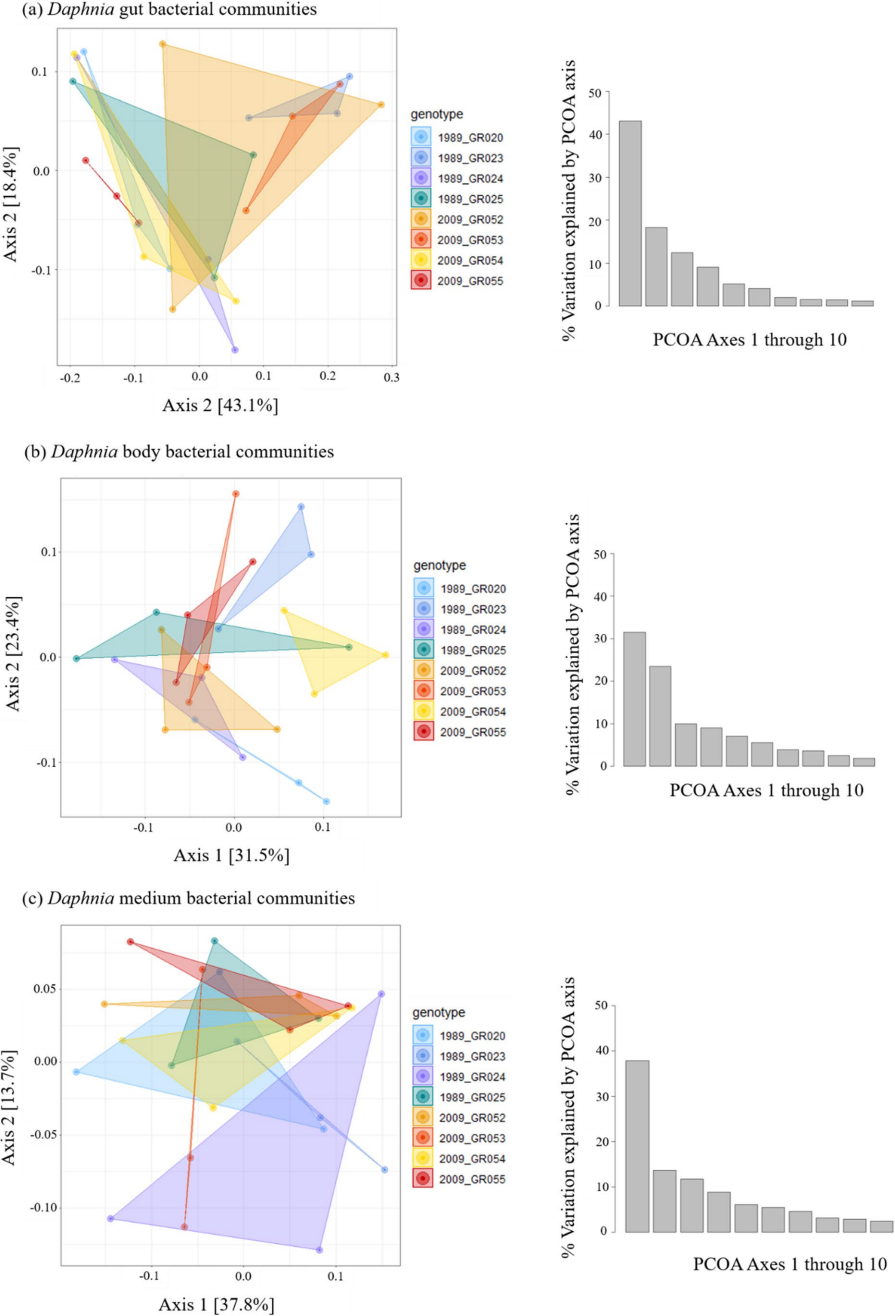
PCOA plots of Weighted Unifrac distance between *Daphnia* **a** gut, **b** body, and **c** medium bacterial communities. Colors represent different genotypes; shades of blue are genotypes originating from 1989; shades of orange/red are those from 2009. Barplots adjacent to PCOA plots show % variation explained by each PCOA axis (Axes 1–10)

The Weighted Unifrac distance varied significantly by genotype but not sediment layer, for both *Daphnia* gut and body bacterial communities (Fig. 1a, b; Table 1). Medium bacterial communities varied neither by genotype nor sediment layer (Fig. 1c; Table 1). For gut bacterial communities, there was overlap between some *Daphnia* genotypes, but certain genotypes, e.g., GR055, GR053, and GR023 cluster separately from the rest (Fig. 1a). For *Daphnia* bodies, most genotypes formed separate clusters (Fig. 1b). We also found 38 ZOTUs that showed abundance distributions skewed towards specific genotypes in the *Daphnia* gut and body tissue. Similarly, 14 ZOTUs were associated with the media of specific *Daphnia* genotypes. However, only two of these ZOTUs were indicative of host genotype in the *Daphnia* gut or body. This suggests that differential abundance of ZOTUs in the media need not correspond with their differential abundance within *Daphnia* (see Fig. S3, Fig. S4 and Table S5).

**Table 1:** PERMANOVAs on Weighted Unifrac distances performed separately for gut (A), body (B), and medium (C) bacterial communities (9999 permutations). *p* < 0.05 are highlighted in bold. % variation column shows the % variation explained based on db-RDA using the *capscale* function

(A)	Gut bacterial communities						% variation
	Df	Sums of sqs	Means sqs	F. model	*R*^2^	*p* value	
Sediment layer	1	0.014	0.0145	0.365	0.013	0.9155	
**Sediment layer:genotype**	**6**	**0.507**	**0.0845**	**2.127**	**0.438**	**0.0079**	
Residuals	16	0.636	0.0397		0.549		
Total	23	1.158			1		45.5
(B)	Body bacterial communities						% variation
	Df	Sums of sqs	Means sqs	F. model	*R*^2^	*p* value	
Sediment layer	1	0.008	0.0082	0.416	0.015	0.916	
**Sediment layer:genotype**	**6**	**0.225**	**0.0374**	**1.898**	**0.41**	**0.006**	
Residuals	16	0.316	0.0197		0.576		
Total	23	0.548			1		43.4
(C)	Medium bacterial communities						% variation
	Df	Sums of sqs	Means sqs	F. model	*R*^2^	*p* value	
Sediment layer	1	0.027	0.0268	0.934	0.042	0.45	
Sediment layer:genotype	6	0.158	0.0264	0.92	0.246	0.59	
Residuals	16	0.459	0.0287		0.713		
Total	23	0.644			1		29.6

### Relationship Between *Daphnia* Genetic Distance and Bacterial Community Composition

**Fig. 2:**
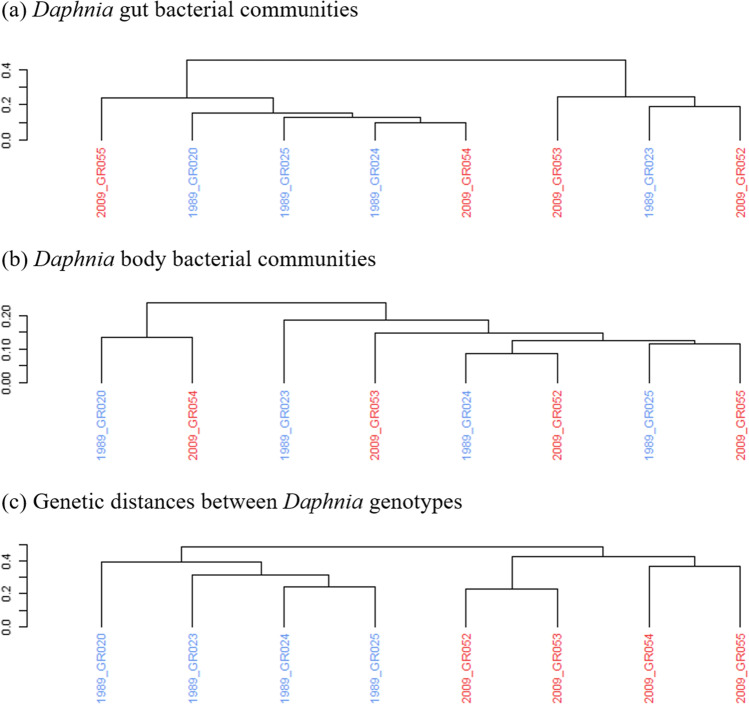
Hierarchical clustering plots of pairwise distance matrices between *Daphnia* genotypes for bacterial communities and host genetic distances; colors represent sediment layer from which genotypes originated (blue–1989, red–2009). **a** Weighted Unifrac distance of average ZOTU counts between *Daphnia* gut bacterial communities, based on 16S sequencing data. **b** Weighted Unifrac distance of average ZOTU counts between *Daphnia* body bacterial communities, based on 16S sequencing data. **c** Genetic distances between *Daphnia* genotypes based on 41,771 SNPs from *Daphnia* whole genome sequencing data. *p* values for correlation between distance matrices were calculated separately using a Mantel test (see Methods)

We further tested for a correlation between the genetic distance among host genotypes based on 41,771 SNPs from whole genome sequencing data and the average Weighted Unifrac distance between the bacterial communities of these genotypes (Table 2). We found no significant correlation between host genetic distance and average Weighted Unifrac distance of bacterial communities at the ZOTU level in *Daphnia* guts (Mantel statistic based on Spearman’s rho *r* =  − 0.21, *p* = 0.889) or *Daphnia* bodies (*r* =  − 0.26, *p* = 0.935). Furthermore, neither *Daphnia* gut nor body bacterial communities clustered by sediment layer of origin, whereas *Daphnia* genotypes did (Fig. 2).

**Table 2:** Mantel test results based on Spearman’s rank correlation (9999 permutations), between Weighted Unifrac distances of Daphnia bacterial communities (based on 16S sequencing data) and genetic distances between Daphnia genotypes (based on whole genome sequencing data)

*Daphnia* tissue compared with genetic distance between genotypes		
(Weighted Unifrac distance of bacterial communities)	*r* statistic	*p* value
*Daphnia* guts	− 0.2124	0.889
*Daphnia* bodies	− 0.266	0.935

### Bacterial Community Composition in *Daphnia* Between Sediment Layers of Origin

**Fig. 3:**
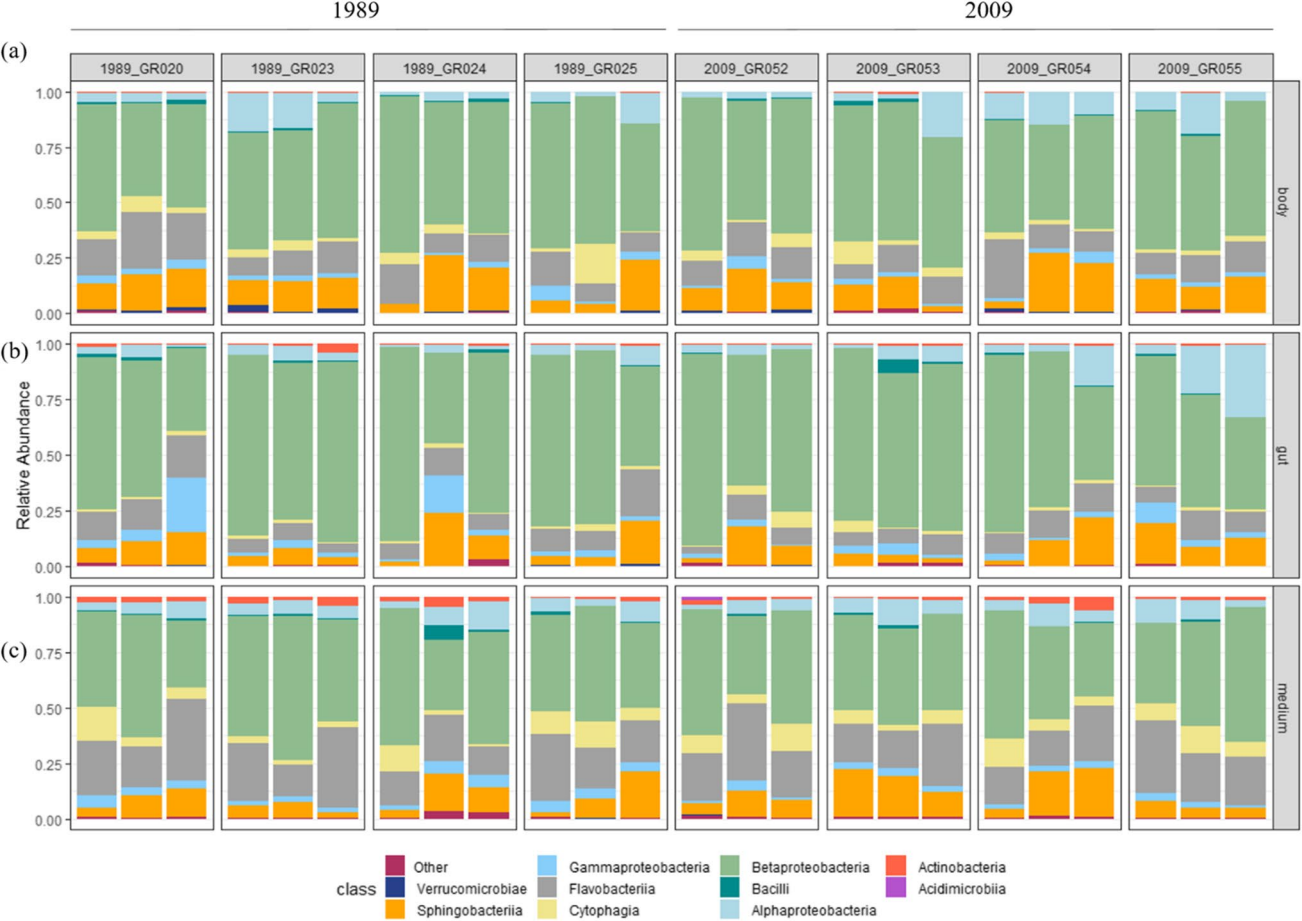
Bacterial communities in *Daphnia* **a** body, **b** gut, and **c** medium. Four columns on the left depict genotypes hatched from 1989 and the four on the right, from 2009. Bacterial classes constituting < 1% of the dataset and not present in every sample are classified as “Other.” The group “Other” is comprised of 23 bacterial classes

The *Daphnia* gut, body, and medium bacterial communities were comprised of 10 dominant classes of bacteria (Fig. 3). Comparisons between sediment layers were made separately for each sample type and averaged over genotypes (see Methods). Alphaproteobacteria was significantly more abundant in the guts of genotypes hatched from 2009 (9.01 ± 9.8%) compared to the guts of those from 1989 (3.89 ± 2.35%) while Acidimicrobiia was significantly more abundant in the bodies of *Daphnia* from 1989 (0.03 ± 0.02%) compared to the bodies of *Daphnia* from 2009 (0.01 ± 0.005%) (Supplementary Table 1). However, the higher abundance of Alphaproteobacteria in genotypes from 2009 is due to higher abundance in a single genotype, 2009_GR055 (Fig. 3) indicating that variation is primarily between *Daphnia* genotypes. Class Acidimicrobiia was rare within *Daphnia* tissue.

### Bacterial Community Composition Between *Daphnia* Tissue and Medium

The most abundant class, Betaproteobacteria, was significantly more abundant in the *Daphnia* gut (65.9 ± 15.6%, mean ± standard deviation) and body (56.6 ± 7.8%) than in the medium (46.5 ± 9.8). The next most abundant class, Flavobacteriia, was significantly more abundant in the medium (22.4 ± 7.3%) than in the *Daphnia* gut (9.8 ± 4.2%), with the *Daphnia* body having an intermediate relative abundance (13.4 ± 5.2). Sphingobacteriia was more abundant in the *Daphnia* body (13.7 ± 6.9%) than in the medium (10.8 ± 6.6%). The less common classes were also differentially distributed across *Daphnia* tissue: Gammaproteobacteria was significantly more abundant in the *Daphnia* gut than the body and medium. Alphaproteobacteria and Verrucomicrobiae were significantly more abundant in the *Daphnia* body than both the gut and medium, whereas Cytophagia, Actinobacteria and Acidimicrobiia were significantly more abundant in the medium than the *Daphnia* body and gut (Fig. 3, Supplementary Tables 2 and 3). We also found 73 ZOTUs that showed abundance distributions skewed towards specific sample types (see Fig. S5 and Table S6).

### Alpha Diversity of Bacterial Communities Between *Daphnia* Genotypes and Tissues

**Fig. 4:**
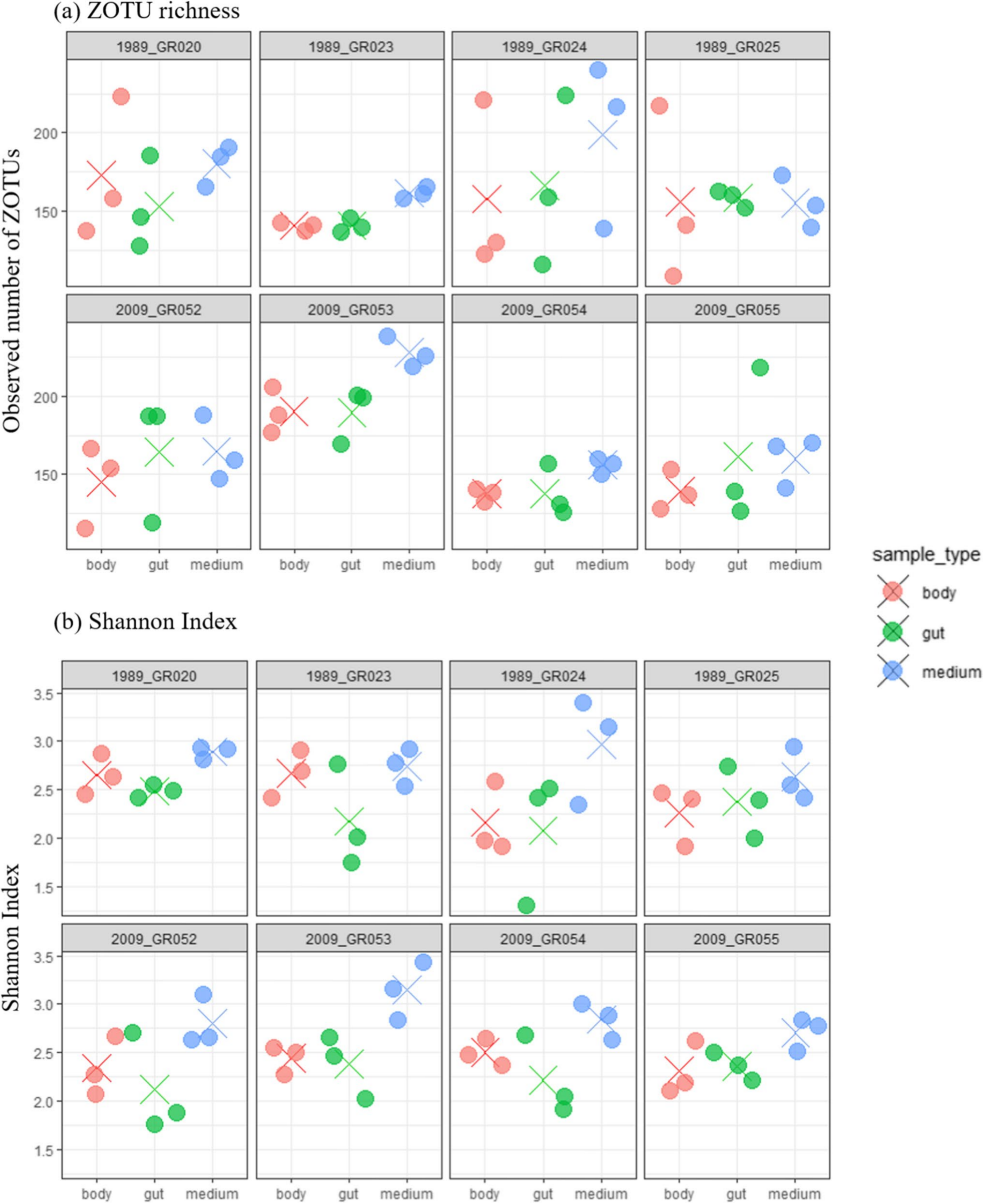
Alpha diversity of bacterial communities in *Daphnia* tissues and media. Each replicate is represented with a colored circle (gut-green, body-red, or medium-blue), and the cross signs represent arithmetic means. Top row includes genotypes originating from sediment layer 1989, bottom rows are those from 2009. **a** ZOTU richness of *Daphnia* gut, body, and medium bacterial communities

We used ZOTU richness and Shannon Index as measures of alpha diversity in the samples (Fig. 4). There was no significant variation in ZOTU richness or Shannon Index between sediment layers (Table 3). ZOTU richness varied significantly across genotypes and sample types. Genotype GR053 had generally higher ZOTU richness, compared to bacterial communities of GR023, GR052, GR054, and GR055 (Supplementary Table 4). Tukey HSD pairwise comparisons showed that the ZOTU richness was higher in medium bacterial communities than the *Daphnia* body though only marginally significant (Supplementary Table 4). The Shannon Index (Fig. 4b) varied significantly only by sample type (Table 3); it was significantly higher for medium than for gut or body bacterial communities (Fig. 4, Supplementary Table 4) indicating that bacterial communities in the medium are more even in composition compared to *Daphnia* body and gut. Notably, ZOTU richness varied by genotype but the Shannon Index did not, suggesting that the differing ZOTUs across genotypes were likely rare in the dataset.

**Table 3:** ANOVA of alpha diversity metrics across sediment layers, genotypes, and sample types. Genotypes are nested within sediment layer. Model used was variable ~ sediment layer/genotype + sample type. ANOVA, *p* < 0.05 are highlighted in bold

ZOTU richness					
	Df	Sums of Sqs	Means Sqs	*F* value	*p* value
Sediment layer	1	110	110	0.14	0.70640
**Sample type**	**2**	**5676**	**2838**	**3.69**	**0.03049**
**Sediment layer:genotype**	**6**	**22,096**	**3683**	**4.79**	**0.00045**
Residuals	62	47,628	768		
Shannon Index					
	Df	Sums of Sqs	Means Sqs	*F* value	*p* value
Sediment layer	1	0	0.001	0.01	0.93000
**Sample type**	**2**	**4.19**	**2.093**	**20.86**	**0.00000**
Sediment layer:genotype	6	0.72	0.119	1.19	0.32000
Residuals	62	6.22	0.1		

## Discussion

We compared the bacterial communities of *Daphnia galeata* guts, bodies, and culturing medium across genotypes simultaneously hatched from two different sediment layers of the same sediment core of Greifensee, and maintained in the laboratory under identical conditions for 5 years before collection of this 16S sequencing data. *Daphnia* genotypes hatched from the two sediment layers (1989 and 2009) formed distinct genetic clusters [29].We found that beta diversity of the gut and body bacterial communities differed significantly between genotypes, confirming our hypothesis that the bacterial community composition has a host-genetic component. However, this variation in beta diversity of bacterial communities did not correlate with genetic distance between their host genotypes. ZOTU richness varied by host genotype (but not by sediment layer of origin), mainly due to one divergent genotype, GR053 (Fig. 4), but the Shannon Index did not vary by genotype or sediment layer of origin. Overall, *Daphnia *bacterial community composition reported in this study is similar to those found in other *Daphnia* studies, particularly the dominance of Betaproteobacteria in *Daphnia* guts and Actinobacteria in the medium [22, 24, 26].

Previous laboratory studies have reported variation in *Daphnia* bacterial communities by genotype, but the extent of host genotype influence differed from study to study. For instance, *D. magna* gut bacterial communities did not vary by genotype in a mesocosm study [24]; similarly, in a laboratory study, whole *D. magna* bacterial communities did not vary by genotype but only by pond of origin [23]. In another experiment, *D. magna* genotype strongly influenced alpha and beta diversities of assembled bacterial communities when the genotypes were made germ-free first. However, the genotype effect was mainly driven by a large divergence between two specific genotypes that also originated from different ponds [26], consistent with a pond-of-origin effect reported elsewhere [23, 25]. When environmental factors such as temperature [25], diet [26], and the composition of environmental bacterial communities [22] were varied, all were found to play a significant role (comparable to or greater than host genotype) in shaping *Daphnia* bacterial communities. In contrast, our study compares bacterial communities of *D. galeata* genotypes originating from the same lake, replicated for genotype and distinct host genetic clusters, and we find that genotypes harbor both compositionally and phylogenetically distinct bacterial communities after being reared in a common garden for 5 years. We also found host genotypes belonging to distinct genetic clusters do not have divergent bacterial communities. Additionally, we empirically validated the common garden in the present study; bacterial communities in the culturing medium did not vary in alpha or beta diversity while *Daphnia*-associated bacterial communities did.

*D. galeata* genotypes isolated from distinct sediment layers in our study (1989 and 2009) differed compositionally in the abundance of some bacterial taxa. Specifically, the guts of *Daphnia* from 2009 contain a higher relative abundance of Alphaproteobacteria than the guts of those from 1989. However, there is no difference in alpha or beta diversity between sediment layers, and the higher relative abundance of Alphaproteobacteria in the guts of 2009 *Daphnia* is due to their higher relative abundance in a single *D. galeata* genotype, GR055 (Fig. 3). Thus, there is no consistency in bacterial communities associated with *D. galeata* genotypes belonging to two distinct host genetic clusters.

Despite the significant variation by host genotype, bacterial community composition in *Daphnia* guts and bodies did not correlate with genetic distance between the host genotypes [29] which is consistent with the absence of variation in bacterial communities of the different *Daphnia* genotypes between sediment layers of origin. In contrast, other aquatic hosts, e.g., sticklebacks [44] and sponges, [45] exhibit variation in bacterial communities between host genotypes and significant positive correlation between host genetic distance and divergence in bacterial community composition. Our results suggest that factors other than host genetics and environmental exposure may shape the structure of host bacterial communities. These could include stochastic processes [46] or interspecies interactions between members of the host’s bacterial community, which may range from co-operative to competitive [47]. Specific dispersal abilities of microbes may also determine their abundance within hosts [48].

The *Daphnia* body bacterial community in our study reflects bacterial groups associated with the filtering apparatus as well as epibionts on the *Daphnia* carapace. Bacterial communities isolated from *Daphnia* body also have differentially abundant associated taxa compared to the gut. Verrucomicrobiae associated with polysaccharide degradation [49] was significantly more abundant in the *Daphnia* body than both the gut and the medium (see Supplementary Tables 2 and 3). Thus, further “omics” studies targeting the transcriptome and metabolome would be required to assess the putative functional roles of such bacterial taxa associated differentially across *Daphnia* tissue [50, 51].

Several studies have demonstrated beneficial effects of *Daphnia*-associated bacteria such as tolerance to cyanobacteria [17], contribution to host development [19], and their general requirement for survival [52]. However, investigation on the adaptive significance and possible co-evolution between *Daphnia* and bacterial communities has yielded mixed results. One study reported no apparent benefit of long-term symbiosis with bacteria in *Daphnia*, or in other species of freshwater zooplankton such as various rotifers and crustaceans [53]. In contrast, *D. magna* benefit more from receiving sympatric vs. allopatric bacterial communities when exposed to environmental stressors such as toxic cyanobacteria [18] though such fitness benefits are also reportedly weaker in semi-natural settings [24]. Furthermore, it has also been suggested that zooplankton-associated bacteria essential to the host are functionally redundant [21, 53]. In this study, we show that while there is variation by host genotype in host-associated bacterial communities, this variation is not determined by genetic distances between hosts. If host-associated bacterial communities are beneficial and required for survival but also functionally redundant, the purpose of this genotype-specific diversity and the mechanism of co-evolution with bacteria among aquatic hosts remains unknown. Hence, a focus on the functional roles of host-associated bacteria and mechanisms of their vertical transmission in hosts could advance our understanding of their role in host ecology and evolution.

## Supplementary Information

Below is the link to the electronic supplementary material.

Supplementary file1 (DOCX 1345 KB)

## Data Availability

Raw sequence data will be available on GenBank and ZOTU table, and associated files required for statistical analyses will be made publicly available on https://doi.org/10.25678/0005DS.

## Code Availability

R code required to reproduce figures, tables, and statistical results together with ZOTU table, and associated files will be publicly available on https://doi.org/10.25678/0005DS upon publishing. Whole-genome sequence data of *Daphnia galeata* genotypes will be publicly available on https://doi.org/10.25678/0005KZ
