# Supplementary material for: Host-Associated Bacterial Communities Vary Between Daphnia galeata Genotypes but Not by Host Genetic Distance
Source: Microb Ecol. 2022 Apr 29;85(4):1578–89. doi: 10.1007/s00248-022-02011-x (PMC10167167; doi:10.1007/s00248-022-02011-x)
Supplement: Supplementary file 1 — Supplementary file1 (DOCX 1345 KB) [file 248_2022_2011_MOESM1_ESM.docx]

Supplementary Data

Host-associated bacterial communities vary between *Daphnia galeata* genotypes but not by host genetic distance

Amruta Rajarajan^1*^, Justyna Wolinska^2, 3^, Jean-Claude Walser^4^, Stuart R. Dennis^1^, Piet Spaak^1^

^1^Swiss Federal Institute of Aquatic Science and Technology (Eawag), Department of Aquatic Ecology, Dübendorf, Switzerland

^2^Freie Universität Berlin (FU), Berlin, Department of Biology, Chemistry, Pharmacy, Institut für Biologie, Germany

^3^Leibniz Institute of Freshwater Ecology and Inland Fisheries (IGB), Berlin, Department of Ecosystem Research, Germany

^4^Genetic Diversity Centre, ETH Zürich, Switzerland

*Corresponding Email: [amruta.rajarajan@eawag.ch/](mailto:amruta.rajarajan@eawag.ch/) [amrutarajarajan@gmail.com](mailto:amrutarajarajan@gmail.com)

Keywords: 16S rDNA, bacteria, Cladocera, microbiome, zooplankton


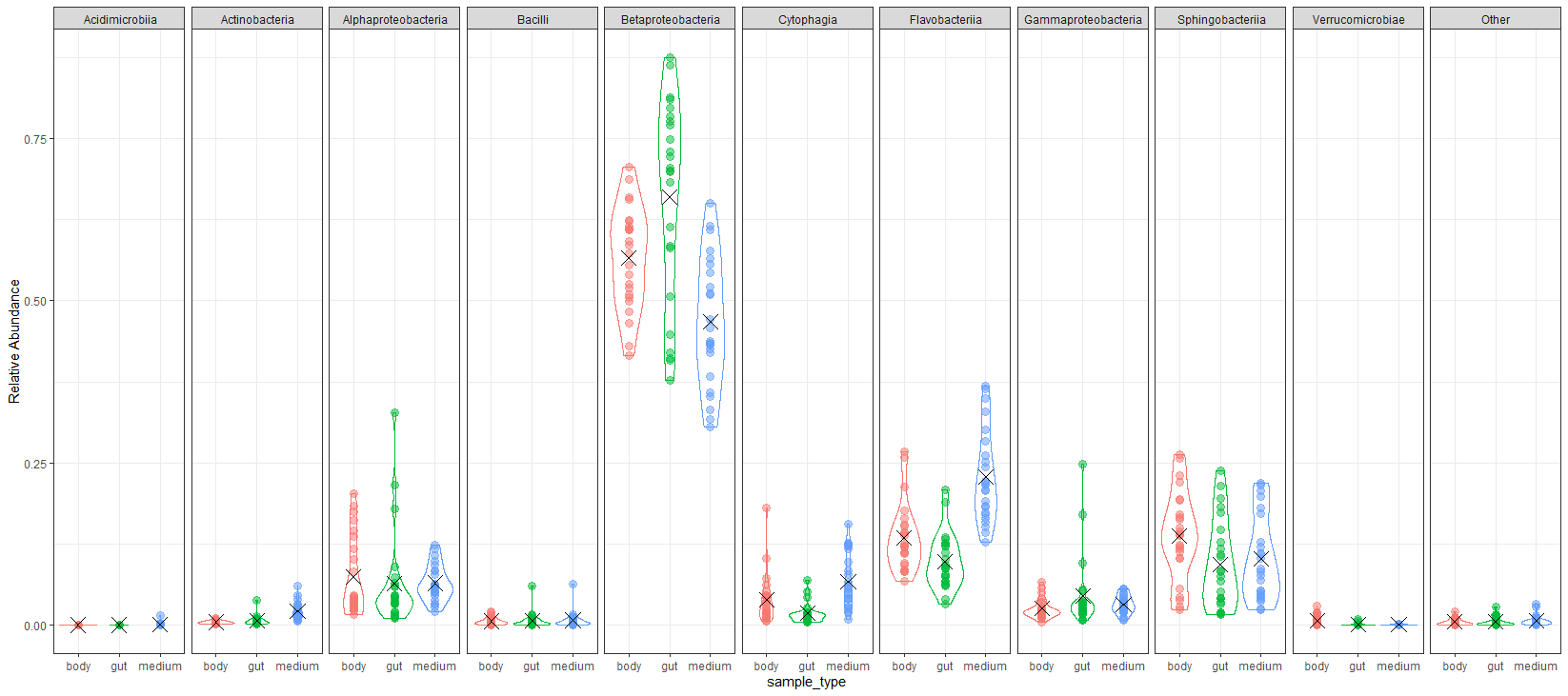


**Suppl. Fig 1:** Relative abundance distributions of the ten dominant bacterial classes across *Daphnia* tissue (guts or bodies) and medium. Black crosses represent mean relative abundance.
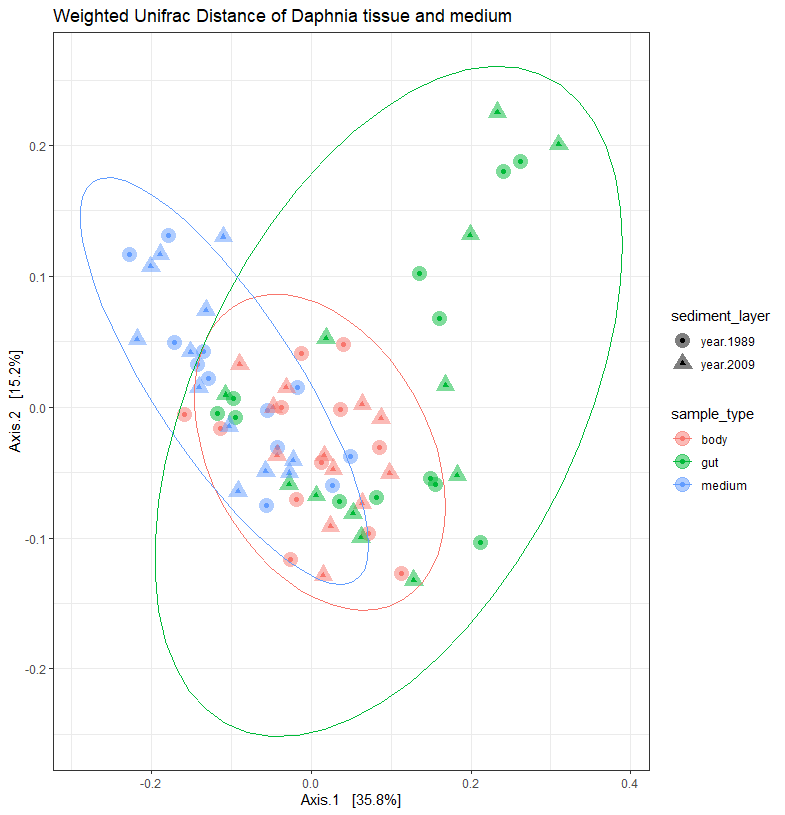


**Suppl. Fig 2:** PCOA of weighted Unifrac distances between *Daphnia* tissue types and medium. Colours represent sample type (*Daphnia* gut, body or medium) and shapes represent the corresponding sediment layer from which genotypes were isolated.


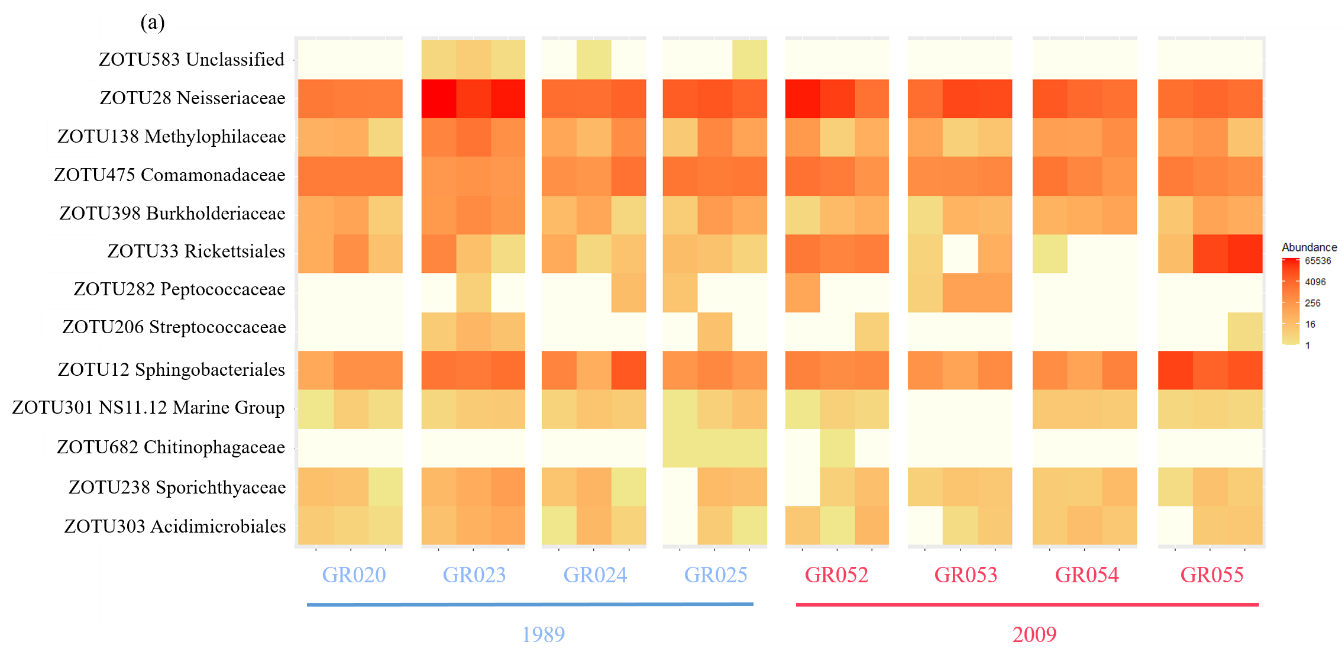


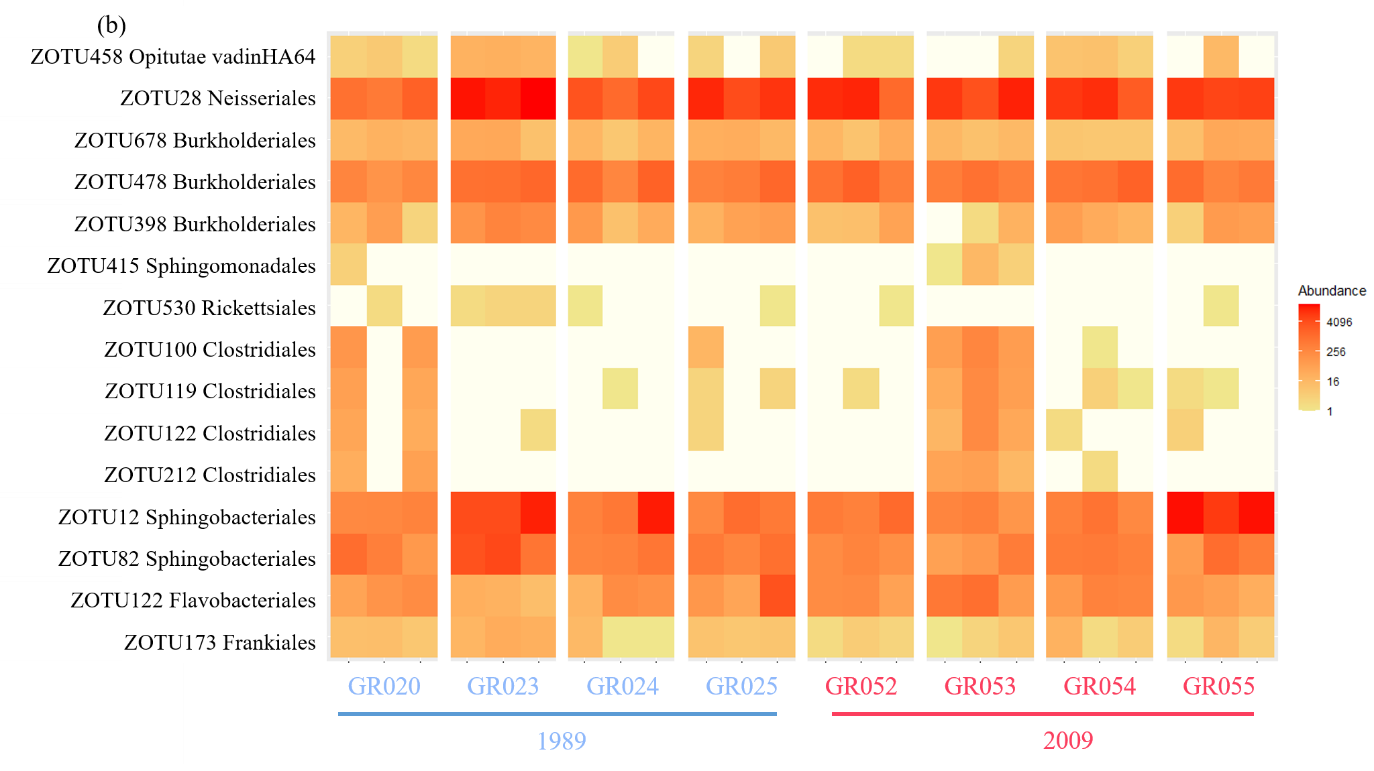

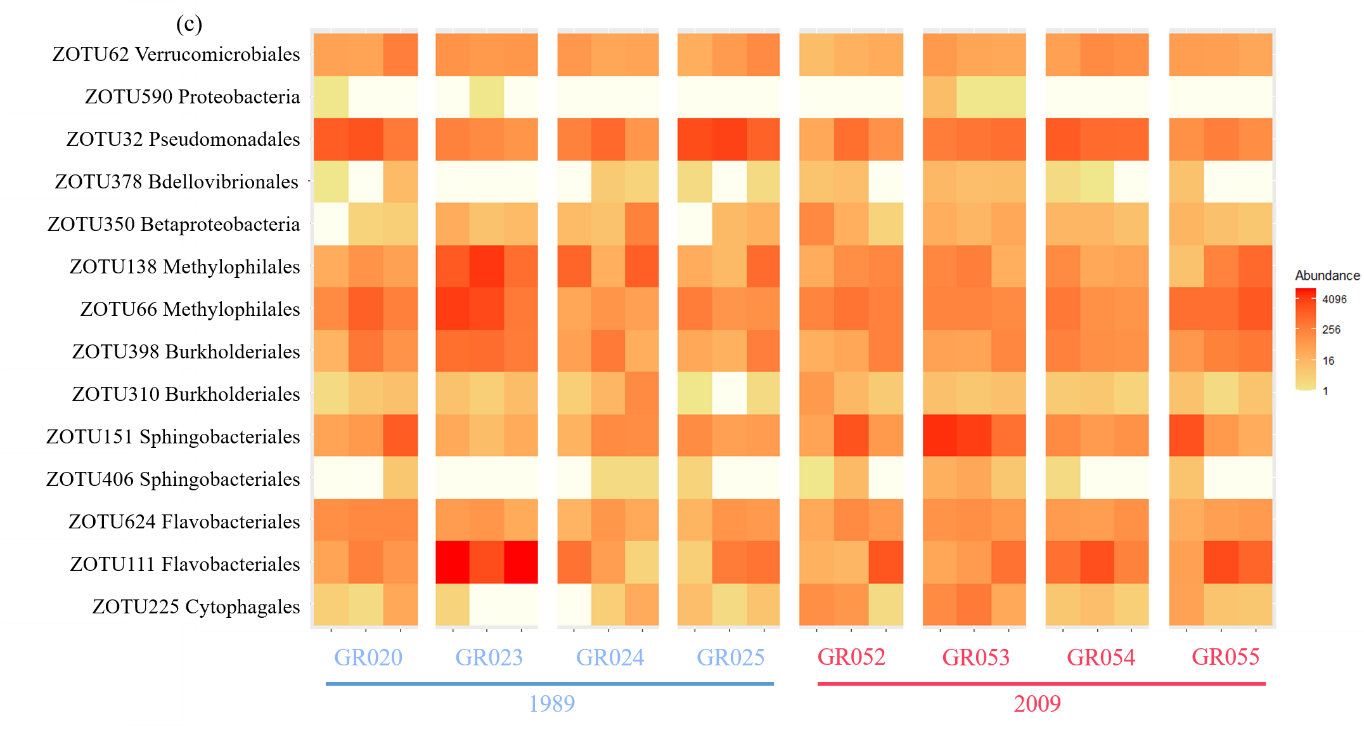


**Suppl. Fig 3:** Heatmap of log-transformed ZOTU counts that were identified as indicative of specific genotypes in Daphnia (a) guts, (b) bodies and (c) medium bacterial communities with their Family (or lowest known taxonomic level, if the ZOTU remains unclassified at the Family level). (psidak < 0.05, see Methods for details on statistical analyses and Table S5 for detailed taxonomic classification of ZOTUs).


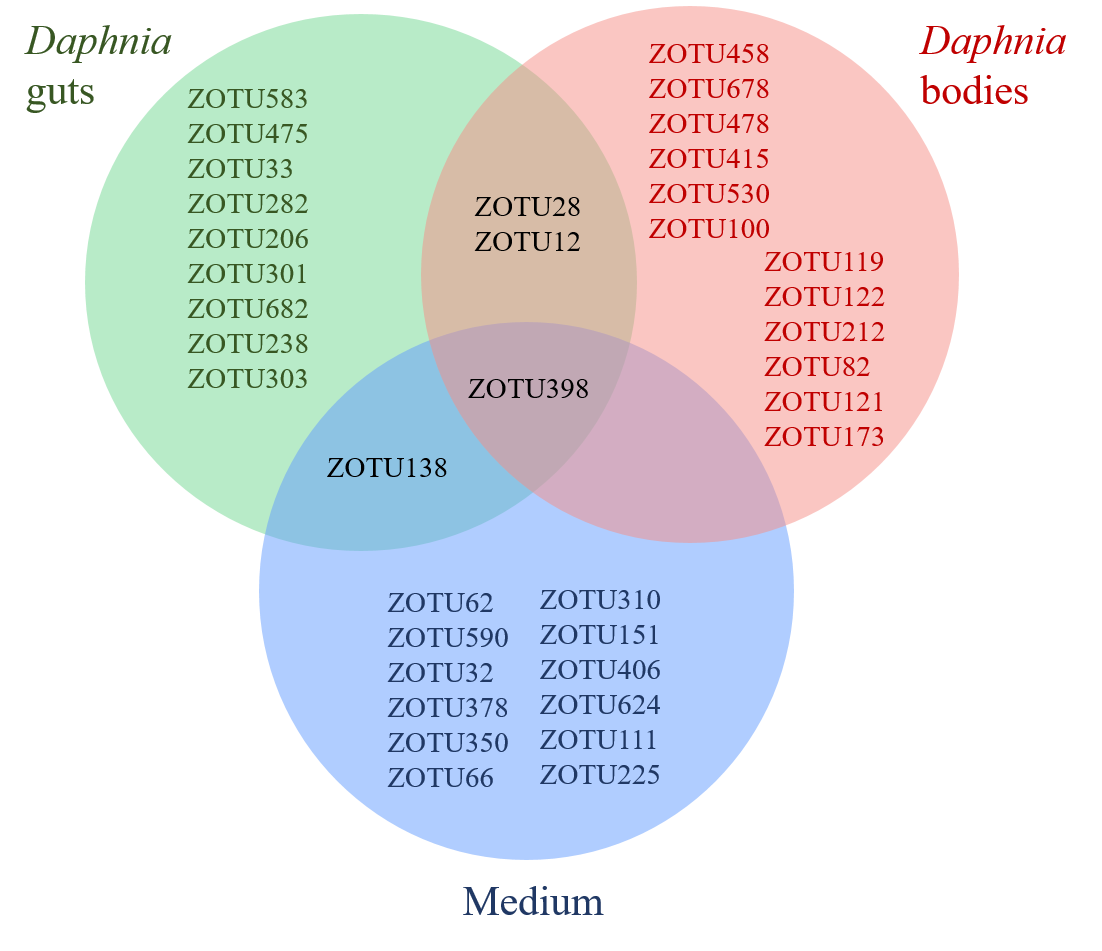


**Suppl. Fig 4:** Venn diagram showing indicator taxa (ZOTUs) specific to Daphnia genotypes among guts (green), body (red) and medium (blue) (psidak < 0.05, see Methods for details on statistical analyses and Fig. S3, Table S5 for more details on these ZOTUs).


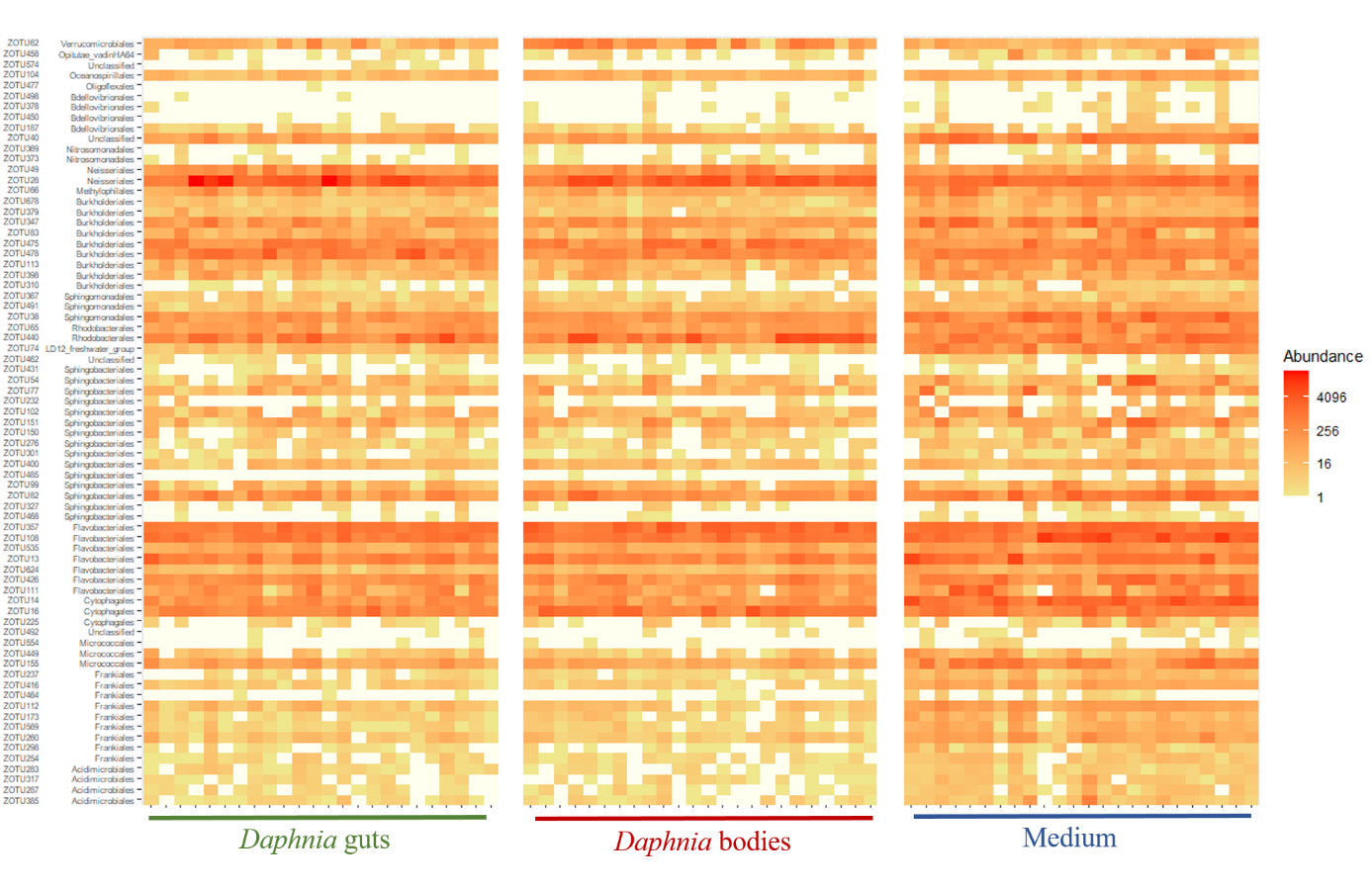


**Suppl. Fig 5:** Heatmap of log-transformed ZOTU counts identified as indicative of Daphnia tissue or medium with their Family (or lowest known taxonomic level, if the ZOTU remains unclassified at the Family level) (psidak < 0.05, see Methods for details on statistical analyses and Table S6 for detailed taxonomic classification of ZOTUs).

| **Bacterial Class** | **Sample Type** | **% Compositional mean ± std dev in *Daphnia* tissue and medium across sediment layer** | |  | **Wald test statistics (2009 vs 1989)** | | | |
| --- | --- | --- | --- | --- | --- | --- | --- | --- |
|  |  |  |  |  |  | |  |  |
|  |  | **1989** | **2009** |  | **Wald stat** | ***p-*adjusted** | |  |
|  |  |  |  |  |  |  | |  |
| Acidimicrobiia | gut | 0.036 ± 0.022 | 0.02 ± 0.01 |  | 1.195745 | 0.5099516 | |  |
|  | **body** | **0.03 ± 0.02** | **0.01 ± 0.005** |  | 2.895135 | 0.041689* | |  |
|  | medium | 0.09 ± 0.07 | 0.18 ± 0.39 |  | 0.24566 | 0.880587 | |  |
| Actinobacteria | gut | 0.86 ± 1.01 | 0.53 ± 0.3 |  | 1.424307 | 0.4456834 | |  |
|  | body | 0.46 ± 0.21 | 0.43 ± 0.27 |  | 0.20475 | 0.9215504 | |  |
|  | medium | 2.37 ± 1.14 | 1.95 ± 1.44 |  | 0.342508 | 0.880587 | |  |
| Alphaproteobacteria | **gut** | **3.89 ± 2.35** | **9.01 ± 9.8** |  | 2.991924 | 0.030494* | |  |
|  | body | 6.52 ± 5.71 | 8.43 ± 6.56 |  | 1.538456 | 0.4544359 | |  |
|  | medium | 6.36 ± 2.7 | 6.6 ± 3.02 |  | 1.355395 | 0.630658 | |  |
| Bacilli | gut | 0.57 ± 0.56 | 0.91 ± 1.67 |  | 1.04808 | 0.5138789 | |  |
|  | body | 0.61 ± 0.55 | 0.62 ± 0.62 |  | 0.88384 | 0.9215504 | |  |
|  | medium | 1.05 ± 1.7 | 0.48 ± 0.49 |  | 1.202086 | 0.630658 | |  |
| Betaproteobacteria | gut | 66.71 ± 16.93 | 65.13 ± 14.85 |  | 0.307291 | 0.758622 | |  |
|  | body | 56.13 ± 8.77 | 57.14 ± 7.13 |  | 0.098481 | 0.9215504 | |  |
|  | medium | 47.67 ± 10.91 | 45.78 ± 9.12 |  | 0.439906 | 0.880587 | |  |
| Cytophagia | gut | 1.4 ± 0.65 | 2.23 ± 2.1 |  | 1.857029 | 0.3481883 | |  |
|  | body | 4.45 ± 4.72 | 3.33 ± 2.67 |  | 0.238921 | 0.9215504 | |  |
|  | medium | 6.15 ± 4.87 | 7.35 ± 3.45 |  | 0.909926 | 0.685951 | |  |
| Flavobacteriia | gut | 10.75 ± 5.15 | 8.86 ± 3.03 |  | 0.912374 | 0.5138789 | |  |
|  | body | 13.9 ± 5.68 | 12.87 ± 4.95 |  | 0.124031 | 0.9215504 | |  |
|  | medium | 22.74 ± 8.22 | 22.85 ± 6.36 |  | 0.888717 | 0.685951 | |  |
| Gammaproteobacteria | gut | 5.77 ± 7.34 | 3.23 ± 2.38 |  | 1.398154 | 0.4456834 | |  |
|  | body | 2.66 ± 1.67 | 2.45 ± 1.4 |  | 0.527856 | 0.9215504 | |  |
|  | medium | 3.9 ± 1.45 | 2.53 ± 1.13 |  | 1.900164 | 0.465718 | |  |
| Sphingobacteriia | gut | 9.28 ± 6.95 | 9.35 ± 6.95 |  | 0.47201 | 0.7136916 | |  |
|  | body | 13.83 ± 7.02 | 13.65 ± 7.03 |  | 0.674431 | 0.9215504 | |  |
|  | medium | 8.83 ± 5.86 | 11.6 ± 7 |  | 1.724177 | 0.465718 | |  |
| Verrucomicrobiae | gut | 0.15 ± 0.26 | 0.09 ± 0.15 |  | 0.889508 | 0.5138789 | |  |
|  | body | 0.93 ± 0.91 | 0.48 ± 0.6 |  | 0.630079 | 0.9215504 | |  |
|  | medium | 0.08 ± 0.05 | 0.06 ± 0.05 |  | 0.150225 | 0.880587 | |  |
| Other | gut | 0.57 ± 0.8 | 0.62 ± 0.65 |  | 0.455415 | 0.7136916 | |  |
|  | body | 0.42 ± 0.39 | 0.57 ± 0.62 |  | 1.646815 | 0.4544359 | |  |
|  | medium | 0.78 ± 1.06 | 0.63 ± 0.43 |  | 0.345263 | 0.880587 | |  |

**Suppl. Table 1** % Compositional mean ± standard deviation of the ten dominant bacterial classes across sediment layers. Bold numbers indicate bacterial classes significantly different between sediment layers based on the Wald test with *p*-values corrected for multiple comparison using the “fdr” method (**p-*adj < 0.05).

| **CLASS** | **GUT** | **BODY** | **MEDIUM** |
| --- | --- | --- | --- |
| Acidimicrobiia | 0.03 ± 0.02^a^ | 0.02 ± 0.02^a^ | **0.13 ± 0.28^b^** |
| Actinobacteria | 0.7 ± 0.75^a^ | 0.45 ± 0.24^b^ | **2.16 ± 1.29^c^** |
| Alphaproteobacteria | 6.45 ± 7.45^a^ | **7.48 ± 6.09^a^** | 6.48 ± 2.8^b^ |
| Bacilli | 0.74 ± 1.23^a^ | 0.62 ± 0.57^a^ | 0.77 ± 1.26^a^ |
| Betaproteobacteria | **65.92 ± 15.6^a^** | 56.64 ± 7.84^a^ | 46.72 ± 9.88^b^ |
| Cytophagia | 1.82 ± 1.58^a^ | 3.89 ± 3.8^b^ | **6.75 ± 4.17^b^** |
| Flavobacteriia | 9.8 ± 4.24^a^ | 13.41 ± 5.24^a,b^ | **22.79 ± 7.18^b^** |
| Gammaproteobacteria | **4.5 ± 5.49^a^** | 2.55 ± 1.51^b^ | 3.21 ± 1.45^b^ |
| Sphingobacteriia | 9.32 ± 6.8^a^ | **13.74 ± 6.87^a^** | 10.21 ± 6.47^b^ |
| Verrucomicrobiae | 0.12 ± 0.21^a^ | **0.7 ± 0.79^b^** | 0.07 ± 0.04^c^ |
| Other | 0.6 ± 0.71^a^ | 0.5 ± 0.51^a^ | 0.71 ± 0.8^a^ |

**Suppl. Table 2** % Compositional mean ± standard deviation of the ten dominant bacterial classes across sample type (*Daphnia* gut, *Daphnia* body or medium). The numbers show the average composition of bacterial classes over the eight genotypes in the study. Different superscript alphabets show statistically different groups across *Daphnia* tissue types and medium based on the Wald test (*p* < 0.05), (see Suppl. Table 2 for all statistical results including *p* values). Bold numbers indicate the sample type in which the bacterial class was most abundant.

| **Bacterial Class** | ***Daphnia* gut vs body** | | |  | ***Daphnia* gut vs medium** | | |  | ***Daphnia* body vs medium** | | |
| --- | --- | --- | --- | --- | --- | --- | --- | --- | --- | --- | --- |
|  | log2FoldChange | Wald stat | *p-*adjusted |  | log2FoldChange | Wald stat | *p-*adjusted |  | log2FoldChange | Wald stat | *p-*adjusted |
| Acidimicrobiia | 0.6140623 | 2.2415370 | 0.05498 |  | -0.6218590 | -2.3539700 | 0.02270* |  | -1.2359210 | -4.6148040 | 0.00001* |
| Actinobacteria | 0.7713312 | 3.3340830 | 0.00314* |  | -1.0605980 | -4.5979900 | 0.00002* |  | -1.8319290 | -7.9321560 | 0.00000* |
| Alphaproteobacteria | -0.0301364 | -0.1069160 | 0.91486 |  | 0.7258110 | 2.5749300 | 0.01576* |  | 0.7559470 | 2.6819060 | 0.01342 |
| Bacilli | 0.3685799 | 0.8839650 | 0.41439 |  | 0.6715560 | 1.6108000 | 0.11795 |  | 0.3029760 | 0.7266430 | 0.57132 |
| Betaproteobacteria | 0.4050682 | 1.9973360 | 0.08395 |  | 1.1781780 | 5.8094000 | 0.00000* |  | 0.7731100 | 3.8120570 | 0.00030* |
| Cytophagia | -1.1142553 | -3.1168130 | 0.00503* |  | -1.2393160 | -3.4668000 | 0.00116* |  | -0.1250600 | -0.3499050 | 0.79426 |
| Flavobacteriia | -0.2451238 | -1.2820620 | 0.27328 |  | -0.5222190 | -2.7315400 | 0.01156* |  | -0.2770950 | -1.4494660 | 0.23133 |
| Gammaproteobacteria | 1.0670655 | 4.0969700 | 0.00023* |  | 1.2703230 | 4.8778600 | 0.00001* |  | 0.2032570 | 0.7803020 | 0.57132 |
| Sphingobacteriia | -0.4297557 | -1.6149710 | 0.16707 |  | 0.6438790 | 2.4195300 | 0.02137* |  | 1.0736350 | 4.0346140 | 0.00015* |
| Verrucomicrobiae | -2.1296778 | -4.9045320 | 0.00001* |  | 1.6394420 | 3.7539300 | 0.00048* |  | 3.7691200 | 8.6536110 | 0.00000* |
| Other | 0.4556065 | 1.2170390 | 0.27328 |  | 0.5532210 | 1.4783000 | 0.13933 |  | 0.0976150 | 0.2607840 | 0.79426 |

**Suppl. Table 3** Pairwise comparison of differential abundance in dominant bacterial classes across *Daphnia* gut, body and medium bacterial communities using Wald test in the DESeq2 package. *p*adj refers to *p*-values adjusted for multiple comparisons using the "fdr" method, significant *p-*adj are indicated with *.

| (A) | **OTU RICHNESS** | | | |
| --- | --- | --- | --- | --- |
| Host genotype |  |  |  |  |
|  | diff | lwr | upr | *p-*adjusted |
| GR023-GR020 | -21.1111 | -65.9762 | 23.75394 | 0.808422 |
| GR024-GR020 | 5.333333 | -39.5317 | 50.19838 | 0.999941 |
| GR025-GR020 | -12.2222 | -57.0873 | 32.64283 | 0.987971 |
| GR052-GR020 | -10.8889 | -55.7539 | 33.97616 | 0.993961 |
| GR053-GR020 | 33.44444 | -11.4206 | 78.30949 | 0.283261 |
| GR054-GR020 | -25.1111 | -69.9762 | 19.75394 | 0.64043 |
| GR055-GR020 | -15.5556 | -60.4206 | 29.30949 | 0.954049 |
| GR024-GR023 | 26.44444 | -18.4206 | 71.30949 | 0.578986 |
| GR025-GR023 | 8.888889 | -35.9762 | 53.75394 | 0.998294 |
| GR052-GR023 | 10.22222 | -34.6428 | 55.08727 | 0.995899 |
| **GR053-GR023** | **54.55556** | **9.690506** | **99.42061** | **0.007705** |
| GR054-GR023 | -4 | -48.8651 | 40.86505 | 0.999992 |
| GR055-GR023 | 5.555556 | -39.3095 | 50.42061 | 0.999923 |
| GR025-GR024 | -17.5556 | -62.4206 | 27.30949 | 0.915616 |
| GR052-GR024 | -16.2222 | -61.0873 | 28.64283 | 0.942944 |
| GR053-GR024 | 28.11111 | -16.7539 | 72.97616 | 0.502142 |
| GR054-GR024 | -30.4444 | -75.3095 | 14.42061 | 0.39931 |
| GR055-GR024 | -20.8889 | -65.7539 | 23.97616 | 0.816505 |
| GR052-GR025 | 1.333333 | -43.5317 | 46.19838 | 1 |
| **GR053-GR025** | **45.66667** | **0.801617** | **90.53172** | **0.043338** |
| GR054-GR025 | -12.8889 | -57.7539 | 31.97616 | 0.983638 |
| GR055-GR025 | -3.33333 | -48.1984 | 41.53172 | 0.999998 |
| GR053-GR052 | 44.33333 | -0.53172 | 89.19838 | 0.054901 |
| GR054-GR052 | -14.2222 | -59.0873 | 30.64283 | 0.97157 |
| GR055-GR052 | -4.66667 | -49.5317 | 40.19838 | 0.999976 |
| **GR054-GR053** | **-58.5556** | **-103.421** | **-13.6905** | **0.003302** |
| **GR055-GR053** | **-49** | **-93.8651** | **-4.13495** | **0.023338** |
| GR055-GR054 | 9.555556 | -35.3095 | 54.42061 | 0.997305 |
|  |  |  |  |  |
| Sample type |  |  |  |  |
|  | diff | lwr | upr | *p-*adjusted |
| gut-body | 3.958333 | -17.0138 | 24.93051 | 0.891766 |
| medium-body | 20.5 | -0.47217 | 41.47217 | 0.056638 |
| medium-gut | 16.54167 | -4.43051 | 37.51384 | 0.147539 |
|  |  |  |  |  |
| (B) | **SHANNON INDEX** | | | |
| Host genotype |  |  |  |  |
|  | diff | lwr | upr | *p-*adjusted |
| GR023-GR020 | -0.1457 | -0.63897 | 0.34757 | 0.98083 |
| GR024-GR020 | -0.27351 | -0.76678 | 0.219759 | 0.651089 |
| GR025-GR020 | -0.25052 | -0.74379 | 0.242747 | 0.742568 |
| GR052-GR020 | -0.25592 | -0.74918 | 0.237353 | 0.721859 |
| GR053-GR020 | -0.01594 | -0.50921 | 0.477332 | 1 |
| GR054-GR020 | -0.15599 | -0.64926 | 0.33728 | 0.971945 |
| GR055-GR020 | -0.21467 | -0.70794 | 0.278598 | 0.862249 |
| GR024-GR023 | -0.12781 | -0.62108 | 0.365458 | 0.99105 |
| GR025-GR023 | -0.10482 | -0.59809 | 0.388446 | 0.997343 |
| GR052-GR023 | -0.11022 | -0.60349 | 0.383052 | 0.996365 |
| GR053-GR023 | 0.129762 | -0.36351 | 0.623031 | 0.990208 |
| GR054-GR023 | -0.01029 | -0.50356 | 0.482979 | 1 |
| GR055-GR023 | -0.06897 | -0.56224 | 0.424297 | 0.999825 |
| GR025-GR024 | 0.022987 | -0.47028 | 0.516256 | 1 |
| GR052-GR024 | 0.017594 | -0.47568 | 0.510863 | 1 |
| GR053-GR024 | 0.257573 | -0.2357 | 0.750842 | 0.715391 |
| GR054-GR024 | 0.117521 | -0.37575 | 0.61079 | 0.994605 |
| GR055-GR024 | 0.058839 | -0.43443 | 0.552108 | 0.99994 |
| GR052-GR025 | -0.00539 | -0.49866 | 0.487875 | 1 |
| GR053-GR025 | 0.234585 | -0.25868 | 0.727854 | 0.800052 |
| GR054-GR025 | 0.094534 | -0.39874 | 0.587803 | 0.998621 |
| GR055-GR025 | 0.035852 | -0.45742 | 0.529121 | 0.999998 |
| GR053-GR052 | 0.239979 | -0.25329 | 0.733248 | 0.781282 |
| GR054-GR052 | 0.099927 | -0.39334 | 0.593196 | 0.998035 |
| GR055-GR052 | 0.041245 | -0.45202 | 0.534514 | 0.999995 |
| GR054-GR053 | -0.14005 | -0.63332 | 0.353217 | 0.984706 |
| GR055-GR053 | -0.19873 | -0.692 | 0.294535 | 0.903035 |
| GR055-GR054 | -0.05868 | -0.55195 | 0.434587 | 0.999941 |
|  |  |  |  |  |
| Sample type |  |  |  |  |
|  | diff | lwr | upr | *p-*adjusted |
| gut-body | -0.14465 | -0.37523 | 0.085931 | 0.291997 |
| **medium-body** | **0.423555** | **0.192977** | **0.654134** | **0.000152** |
| **medium-gut** | **0.568202** | **0.337624** | **0.798781** | **0.000000** |
|  |  |  |  |  |

**Suppl. Table 4** TukeyHSD for alpha diversity metrics OTU richness (A) and Shannon Index (B), investigating pairwise differences between *Daphnia* genotypes and sample types (*Daphnia* gut, body or medium). *p*<0.05 are indicated in bold.

| (a) | ***Daphnia* guts** | | | | | |
| --- | --- | --- | --- | --- | --- | --- |
|  |  |  |  |  |  |  |
| **ZOTU** | **phylum** | **class** | **order** | **family** | **genus** | **species** |
| ZOTU583 | Unclassified | Unclassified | Unclassified | Unclassified | Unclassified | Unknown |
| ZOTU28 | Proteobacteria | Betaproteobacteria | Neisseriales | Neisseriaceae | *Leeia* | Unknown |
| ZOTU138 | Proteobacteria | Betaproteobacteria | Methylophilales | Methylophilaceae | Unclassified | Unknown |
| ZOTU475 | Proteobacteria | Betaproteobacteria | Burkholderiales | Comamonadaceae | Unclassified | Unknown |
| ZOTU398 | Proteobacteria | Betaproteobacteria | Burkholderiales | Burkholderiaceae | *Polynucleobacter* | Unknown |
| ZOTU33 | Proteobacteria | Alphaproteobacteria | Rickettsiales | Unclassified | Unclassified | Unknown |
| ZOTU282 | Firmicutes | Clostridia | Clostridiales | Peptococcaceae | Unclassified | Unknown |
| ZOTU206 | Firmicutes | Bacilli | Lactobacillales | Streptococcaceae | *Lactococcus* | Unknown |
| ZOTU12 | Bacteroidetes | Sphingobacteriia | Sphingobacteriales | Unclassified | Unclassified | Unknown |
| ZOTU301 | Bacteroidetes | Sphingobacteriia | Sphingobacteriales | NS11.12 marine group | Unclassified | Unknown |
| ZOTU682 | Bacteroidetes | Sphingobacteriia | Sphingobacteriales | Chitinophagaceae | *Flavitalea* | Unknown |
| ZOTU238 | Actinobacteria | Actinobacteria | Frankiales | Sporichthyaceae | Unclassified | Unknown |
| ZOTU303 | Actinobacteria | Acidimicrobiia | Acidimicrobiales | Unclassified | Unclassified | Unknown |
|  |  |  |  |  |  |  |
| (b) | ***Daphnia* bodies** | | | | | |
|  |  |  |  |  |  |  |
| **ZOTU** | **phylum** | **class** | **order** | **family** | **genus** | **species** |
| ZOTU458 | Verrucomicrobia | Opitutae | Opitutae_vadinHA64 | Unclassified | Unclassified | Unknown |
| ZOTU28 | Proteobacteria | Betaproteobacteria | Neisseriales | Neisseriaceae | *Leeia* | Unknown |
| ZOTU678 | Proteobacteria | Betaproteobacteria | Burkholderiales | Unclassified | Unclassified | Unknown |
| ZOTU478 | Proteobacteria | Betaproteobacteria | Burkholderiales | Burkholderiaceae | Unclassified | Unknown |
| ZOTU398 | Proteobacteria | Betaproteobacteria | Burkholderiales | Burkholderiaceae | *Polynucleobacter* | Unknown |
| ZOTU415 | Proteobacteria | Alphaproteobacteria | Sphingomonadales | Sphingomonadaceae | *Sphingomonas* | Unknown |
| ZOTU530 | Proteobacteria | Alphaproteobacteria | Rickettsiales | Candidatus_Odyssella | Unclassified | Unknown |
| ZOTU100 | Firmicutes | Clostridia | Clostridiales | Unclassified | Unclassified | Unknown |
| ZOTU119 | Firmicutes | Clostridia | Clostridiales | Finegoldia | Unclassified | Unknown |
| ZOTU122 | Firmicutes | Clostridia | Clostridiales | Anaerococcus | Unclassified | Unknown |
| ZOTU212 | Firmicutes | Bacilli | Lactobacillales | Aerococcaceae | Unclassified | Unknown |
| ZOTU12 | Bacteroidetes | Sphingobacteriia | Sphingobacteriales | Unclassified | Unclassified | Unknown |
| ZOTU82 | Bacteroidetes | Sphingobacteriia | Sphingobacteriales | Chitinophagaceae | *Sediminibacterium* | Unknown |
| ZOTU121 | Bacteroidetes | Flavobacteriia | Flavobacteriales | Cryomorphaceae | *Fluviicola* | Unknown |
| ZOTU173 | Actinobacteria | Actinobacteria | Frankiales | Sporichthyaceae | Unclassified | Unknown |
|  |  |  |  |  |  |  |
| (c) | **Medium** | | | | | |
|  |  |  |  |  |  |  |
| **ZOTU** | **phylum** | **class** | **order** | **family** | **genus** | **species** |
| ZOTU62 | Verrucomicrobia | Verrucomicrobiae | Verrucomicrobiales | Verrucomicrobiaceae | Unclassified | Unknown |
| ZOTU590 | Proteobacteria | Unclassified | Unclassified | Unclassified | Unclassified | Unknown |
| ZOTU32 | Proteobacteria | Gammaproteobacteria | Pseudomonadales | Pseudomonadaceae | *Pseudomonas* | Unknown |
| ZOTU378 | Proteobacteria | Deltaproteobacteria | Bdellovibrionales | Bdellovibrionaceae | Bdellovibrio | Unknown |
| ZOTU350 | Proteobacteria | Betaproteobacteria | Unclassified | Unclassified | Unclassified | Unknown |
| ZOTU138 | Proteobacteria | Betaproteobacteria | Methylophilales | Methylophilaceae | Unclassified | Unknown |
| ZOTU66 | Proteobacteria | Betaproteobacteria | Methylophilales | Methylophilaceae | Unclassified | Unknown |
| ZOTU398 | Proteobacteria | Betaproteobacteria | Burkholderiales | Burkholderiaceae | *Polynucleobacter* | Unknown |
| ZOTU310 | Proteobacteria | Betaproteobacteria | Burkholderiales | Alcaligenaceae | *MWH.UniP1 aquatic group* | Unknown |
| ZOTU151 | Bacteroidetes | Sphingobacteriia | Sphingobacteriales | Sphingobacteriaceae | *Solitalea* | Unknown |
| ZOTU406 | Bacteroidetes | Sphingobacteriia | Sphingobacteriales | Chitinophagaceae | *Parasegetibacter* | Unknown |
| ZOTU624 | Bacteroidetes | Flavobacteriia | Flavobacteriales | Flavobacteriaceae | *Flavobacterium* | Unknown |
| ZOTU111 | Bacteroidetes | Flavobacteriia | Flavobacteriales | Cryomorphaceae | *Fluviicola* | Unknown |
| ZOTU225 | Bacteroidetes | Cytophagia | Cytophagales | Cyclobacteriaceae | Unclassified | Unknown |
|  |  |  |  |  |  |  |

**Suppl. Table 5** Taxonomic classification of ZOTUs found to be indicative of Daphnia genotype among (a) guts, (b) bodies and (c) medium. psidak < 0.05, see Methods for details on statistical analyses and Fig. S3 for their corresponding abundance distribution plots.

|  | ***Daphnia* tissue/ medium** | | | | | |
| --- | --- | --- | --- | --- | --- | --- |
|  |  |  |  |  |  |  |
| **ZOTU** | **phylum** | **class** | **order** | **family** | **genus** | **species** |
| ZOTU62 | Verrucomicrobia | Verrucomicrobiae | Verrucomicrobiales | Verrucomicrobiaceae | Unclassified | Unknown |
| ZOTU458 | Verrucomicrobia | Opitutae | Opitutae vadinHA64 | Unclassified | Unclassified | Unknown |
| ZOTU574 | Saccharibacteria | Unclassified | Unclassified | Unclassified | Unclassified | Unknown |
| ZOTU104 | Proteobacteria | Gammaproteobacteria | Oceanospirillales | Oceanospirillaceae | *Pseudohongiella* | Unknown |
| ZOTU477 | Proteobacteria | Deltaproteobacteria | Oligoflexales | Oligoflexaceae | Unclassified | Unknown |
| ZOTU498 | Proteobacteria | Deltaproteobacteria | Bdellovibrionales | Bdellovibrionaceae | *Bdellovibrio* | Unknown |
| ZOTU378 | Proteobacteria | Deltaproteobacteria | Bdellovibrionales | Bdellovibrionaceae | *Bdellovibrio* | Unknown |
| ZOTU450 | Proteobacteria | Deltaproteobacteria | Bdellovibrionales | Bdellovibrionaceae | *Bdellovibrio* | Unknown |
| ZOTU187 | Proteobacteria | Deltaproteobacteria | Bdellovibrionales | Bacteriovoracaceae | *Bacteriovorax* | Unknown |
| ZOTU40 | Proteobacteria | Betaproteobacteria | Unclassified | Unclassified | Unclassified | Unknown |
| ZOTU369 | Proteobacteria | Betaproteobacteria | Nitrosomonadales | Nitrosomonadaceae | Unclassified | Unknown |
| ZOTU373 | Proteobacteria | Betaproteobacteria | Nitrosomonadales | Gallionellaceae | Unclassified | Unknown |
| ZOTU49 | Proteobacteria | Betaproteobacteria | Neisseriales | Neisseriaceae | *Leeia* | Unknown |
| ZOTU28 | Proteobacteria | Betaproteobacteria | Neisseriales | Neisseriaceae | *Leeia* | Unknown |
| ZOTU66 | Proteobacteria | Betaproteobacteria | Methylophilales | Methylophilaceae | Unclassified | Unknown |
| ZOTU678 | Proteobacteria | Betaproteobacteria | Burkholderiales | Unclassified | Unclassified | Unknown |
| ZOTU379 | Proteobacteria | Betaproteobacteria | Burkholderiales | Oxalobacteraceae | Unclassified | Unknown |
| ZOTU347 | Proteobacteria | Betaproteobacteria | Burkholderiales | Oxalobacteraceae | Unclassified | Unknown |
| ZOTU83 | Proteobacteria | Betaproteobacteria | Burkholderiales | Oxalobacteraceae | *Massilia* | Unknown |
| ZOTU475 | Proteobacteria | Betaproteobacteria | Burkholderiales | Comamonadaceae | Unclassified | Unknown |
| ZOTU478 | Proteobacteria | Betaproteobacteria | Burkholderiales | Burkholderiaceae | Unclassified | Unknown |
| ZOTU113 | Proteobacteria | Betaproteobacteria | Burkholderiales | Burkholderiaceae | *Limnobacter* | Unknown |
| ZOTU398 | Proteobacteria | Betaproteobacteria | Burkholderiales | Burkholderiaceae | *Polynucleobacter* | Unknown |
| ZOTU310 | Proteobacteria | Betaproteobacteria | Burkholderiales | Alcaligenaceae | *MWH.UniP1 aquatic group* | Unknown |
| ZOTU367 | Proteobacteria | Alphaproteobacteria | Sphingomonadales | Sphingomonadaceae | Unclassified | Unknown |
| ZOTU491 | Proteobacteria | Alphaproteobacteria | Sphingomonadales | Sphingomonadaceae | *Sphingopyxis* | Unknown |
| ZOTU38 | Proteobacteria | Alphaproteobacteria | Sphingomonadales | Sphingomonadaceae | Unclassified | Unknown |
| ZOTU65 | Proteobacteria | Alphaproteobacteria | Rhodobacterales | Rhodobacteraceae | Unclassified | Unknown |
| ZOTU440 | Proteobacteria | Alphaproteobacteria | Rhodobacterales | Rhodobacteraceae | *Rhodobacter* | Unknown |
| ZOTU74 | Proteobacteria | Alphaproteobacteria | LD12 freshwater group | Unclassified | Unclassified | Unknown |
| ZOTU462 | Chloroflexi | SL56 marine group | Unclassified | Unclassified | Unclassified | Unknown |
| ZOTU431 | Bacteroidetes | Sphingobacteriia | Sphingobacteriales | Unclassified | Unclassified | Unknown |
| ZOTU54 | Bacteroidetes | Sphingobacteriia | Sphingobacteriales | Unclassified | Unclassified | Unknown |
| ZOTU77 | Bacteroidetes | Sphingobacteriia | Sphingobacteriales | Unclassified | Unclassified | Unknown |
| ZOTU232 | Bacteroidetes | Sphingobacteriia | Sphingobacteriales | Unclassified | Unclassified | Unknown |
| ZOTU102 | Bacteroidetes | Sphingobacteriia | Sphingobacteriales | Unclassified | Unclassified | Unknown |
| ZOTU151 | Bacteroidetes | Sphingobacteriia | Sphingobacteriales | Sphingobacteriaceae | *Solitalea* | Unknown |
| ZOTU150 | Bacteroidetes | Sphingobacteriia | Sphingobacteriales | Sphingobacteriaceae | *Pedobacter* | Unknown |
| ZOTU276 | Bacteroidetes | Sphingobacteriia | Sphingobacteriales | NS11.12 marine group | Unclassified | Unknown |
| ZOTU301 | Bacteroidetes | Sphingobacteriia | Sphingobacteriales | NS11.12 marine group | Unclassified | Unknown |
| ZOTU400 | Bacteroidetes | Sphingobacteriia | Sphingobacteriales | NS11.12 marine group | Unclassified | Unknown |
| ZOTU485 | Bacteroidetes | Sphingobacteriia | Sphingobacteriales | Chitinophagaceae | Unclassified | Unknown |
| ZOTU99 | Bacteroidetes | Sphingobacteriia | Sphingobacteriales | Chitinophagaceae | Unclassified | Unknown |
| ZOTU82 | Bacteroidetes | Sphingobacteriia | Sphingobacteriales | Chitinophagaceae | *Sediminibacterium* | Unknown |
| ZOTU327 | Bacteroidetes | Sphingobacteriia | Sphingobacteriales | Chitinophagaceae | *Ferruginibacter* | Unknown |
| ZOTU468 | Bacteroidetes | Sphingobacteriia | Sphingobacteriales | Chitinophagaceae | Unclassified | Unknown |
| ZOTU357 | Bacteroidetes | Flavobacteriia | Flavobacteriales | Flavobacteriaceae | *Flavobacterium* | Unknown |
| ZOTU108 | Bacteroidetes | Flavobacteriia | Flavobacteriales | Flavobacteriaceae | *Flavobacterium* | Unknown |
| ZOTU535 | Bacteroidetes | Flavobacteriia | Flavobacteriales | Flavobacteriaceae | *Flavobacterium* | Unknown |
| ZOTU13 | Bacteroidetes | Flavobacteriia | Flavobacteriales | Flavobacteriaceae | *Flavobacterium* | Unknown |
| ZOTU624 | Bacteroidetes | Flavobacteriia | Flavobacteriales | Flavobacteriaceae | *Flavobacterium* | Unknown |
| ZOTU426 | Bacteroidetes | Flavobacteriia | Flavobacteriales | Flavobacteriaceae | *Flavobacterium* | Unknown |
| ZOTU111 | Bacteroidetes | Flavobacteriia | Flavobacteriales | Cryomorphaceae | *Fluviicola* | Unknown |
| ZOTU14 | Bacteroidetes | Cytophagia | Cytophagales | Cytophagaceae | *Pseudarcicella* | Unknown |
| ZOTU16 | Bacteroidetes | Cytophagia | Cytophagales | Cytophagaceae | Unclassified | Unknown |
| ZOTU225 | Bacteroidetes | Cytophagia | Cytophagales | Cyclobacteriaceae | Unclassified | Unknown |
| ZOTU492 | Actinobacteria | Unclassified | Unclassified | Unclassified | Unclassified | Unknown |
| ZOTU554 | Actinobacteria | Actinobacteria | Micrococcales | Microbacteriaceae | *Candidatus Rhodoluna* | Unknown |
| ZOTU449 | Actinobacteria | Actinobacteria | Micrococcales | Microbacteriaceae | *Candidatus Limnoluna* | Unknown |
| ZOTU155 | Actinobacteria | Actinobacteria | Micrococcales | Microbacteriaceae | Unclassified | Unknown |
| ZOTU237 | Actinobacteria | Actinobacteria | Frankiales | Sporichthyaceae | Unclassified | Unknown |
| ZOTU416 | Actinobacteria | Actinobacteria | Frankiales | Sporichthyaceae | Unclassified | Unknown |
| ZOTU464 | Actinobacteria | Actinobacteria | Frankiales | Sporichthyaceae | Unclassified | Unknown |
| ZOTU112 | Actinobacteria | Actinobacteria | Frankiales | Sporichthyaceae | Unclassified | Unknown |
| ZOTU173 | Actinobacteria | Actinobacteria | Frankiales | Sporichthyaceae | Unclassified | Unknown |
| ZOTU569 | Actinobacteria | Actinobacteria | Frankiales | Sporichthyaceae | Unclassified | Unknown |
| ZOTU260 | Actinobacteria | Actinobacteria | Frankiales | Sporichthyaceae | *Candidatus Planktophila* | Unknown |
| ZOTU298 | Actinobacteria | Actinobacteria | Frankiales | Sporichthyaceae | Unclassified | Unknown |
| ZOTU254 | Actinobacteria | Actinobacteria | Frankiales | Sporichthyaceae | Unclassified | Unknown |
| ZOTU283 | Actinobacteria | Acidimicrobiia | Acidimicrobiales | Acidimicrobiaceae | *CL500.29 marine group* | Unknown |
| ZOTU317 | Actinobacteria | Acidimicrobiia | Acidimicrobiales | Acidimicrobiaceae | *CL500.29 marine group* | Unknown |
| ZOTU287 | Actinobacteria | Acidimicrobiia | Acidimicrobiales | Acidimicrobiaceae | *CL500.29 marine group* | Unknown |
| ZOTU385 | Actinobacteria | Acidimicrobiia | Acidimicrobiales | Acidimicrobiaceae | *CL500.29 marine group* | Unknown |
|  |  |  |  |  |  |  |

**Suppl. Table 6** Taxonomic classification of ZOTUs found to be indicative of sample type, i.e. Daphnia gut, body or medium. psidak < 0.05, see Methods for details on statistical analyses and Fig. S4 for their corresponding relative abundance distribution plots.
